# Electrically stimulated in vitro heart cell mimic of acute exercise reveals novel immediate cellular responses to exercise: Reduced contractility and metabolism, but maintained calcium cycling and increased myofilament calcium sensitivity

**DOI:** 10.1002/cbf.3847

**Published:** 2023-09-04

**Authors:** Ana Da Silva Costa, Iffath Ghouri, Alexander Johnston, Karen McGlynn, Andrew McNair, Peter Bowman, Natasha Malik, Johanne Hurren, Tomas Bingelis, Michael Dunne, Godfrey L. Smith, Ole J. Kemi

**Affiliations:** ^1^ School of Cardiovascular and Metabolic Health, College of Medical, Veterinary and Life Sciences University of Glasgow Glasgow UK; ^2^ Graduate School, College of Medical, Veterinary and Life Sciences University of Glasgow Glasgow UK; ^3^ Wellcome Centre for Mitochondrial Research, Translational and Clinical Research Institute, Faculty of Medical Sciences Newcastle University Newcastle upon Tyne UK

**Keywords:** autofluorescence, calcium, cardiomyocyte, cells‐in‐a‐dish, contractility, exercise

## Abstract

Cardiac cellular responses to acute exercise remain undescribed. We present a model for mimicking acute aerobic endurance exercise to freshly isolated cardiomyocytes by evoking exercise‐like contractions over prolonged periods of time with trains of electrical twitch stimulations. We then investigated immediate contractile, Ca^2+^, and metabolic responses to acute exercise in perfused freshly isolated left ventricular rat cardiomyocytes, after a matrix‐design optimized protocol and induced a mimic for acute aerobic endurance exercise by trains of prolonged field twitch stimulations. Acute exercise decreased cardiomyocyte fractional shortening 50%–80% (*p* < .01). This was not explained by changes to intracellular Ca^2+^ handling (*p* > .05); rather, we observed a weak insignificant Ca^2+^ transient increase (*p* = .11), while myofilament Ca^2+^ sensitivity increased 20%–70% (*p* < .05). Acidic pH 6.8 decreased fractional shortening 20%–70% (*p* < .05) because of 20%–30% decreased Ca^2+^ transients (*p* < .05), but no difference occurred between control and acute exercise (*p* > .05). Addition of 1 or 10 mM La^−^ increased fractional shortening in control (1 mM La^−^: no difference, *p* > .05; 10 mM La^−^: 20%–30%, *p* < .05) and acute exercise (1 mM La^−^: 40%–90%, *p* < .01; 10 mM La^−^: 50%–100%, *p* < .01) and rendered acute exercise indifferent from control (*p* > .05). Intrinsic autofluorescence showed a resting NAD_state_ of 0.59 ± 0.04 and FAD_state_ of 0.17 ± 0.03, while acute exercise decreased NADH/FAD ratio 8% (*p* < .01), indicating intracellular oxidation. In conclusion, we show a novel approach for studying immediate acute cardiomyocyte responses to aerobic endurance exercise. We find that acute exercise in cardiomyocytes decreases contraction, but Ca^2+^ handling and myofilament Ca^2+^ sensitivity compensate for this, while acidosis and reduced energy substrate and mitochondrial ATP generation explain this.

## Significance statement

Endurance exercise is taxing to the heart and it leads to cardiac exhaustion and fatigue with contractile impairment. However, a mechanism for this has not yet been found, at least partly because this has been impossible to study in heart muscle cells (cardiomyocytes, where contraction occurs) since the process of ascertaining cells after physical exercise in and of itself uncouples the exercise effect. We took a different approach and came up with a novel protocol; instead of exercising animals and then preparing cells, we first prepared cells and then supplied exercise to the cell. This showed that the reduced contractile function during and immediately after exercise was caused by acidosis and metabolic impairments, while Ca^2+^ lessened the dys‐contraction.

## 1 INTRODUCTION

Cardiac pump capacity is largely determined by cardiomyocyte excitation‐contraction coupling, Ca^2+^ handling and available energy substrate. This is because Ca^2+^ binding to myofilaments initiates and drives contraction‐relaxation cycles and since both contraction–relaxation cycling and the associated Ca^2+^ handling across both cell and sarcoplasmic reticulum (SR) membranes demand energy in the form of adenosine triphosphate (ATP). Mitochondrial oxidative phosphorylation generates the bulk of this, driven by the electron transfer (redox) potential of nicotinamide adenine dinucleotide (NAD) and flavin adenine dinucleotide (FAD), relative to O_2_ availability.^1^

Aerobic endurance exercise training improves a number of cardiac and cardiomyocyte indices of contractile capacity and performance, evidenced by studies in both humans,^2^ and experimental animals.^3^, ^4^, ^5^, ^6^, ^7^, ^8^ In these studies, regular exercise training improves cardiomyocyte contractility as measured during resting basal conditions as well as during increased heart rates and stimulation frequencies that occur during the actual exercise, and this is typically superseded by improved intracellular transmembrane and SR Ca^2+^ handling,^4^, ^5^, ^9^ increased myofilament Ca^2+^ sensitization and force generation,^3^, ^10^ faster diastolic SR re‐uptake of Ca^2+^,^8^ as well as metabolic adaptations that enhance generation of ATP^11^; hence, the key determinants of cardiac pump capacity.

The improvement to aerobic endurance exercise training described above effectively occurs because of an accumulated adaptation to the repeated individual acute exercise bouts. The stimulus is that each time an acute exercise bout arrives, the heart responds by transiently increasing heart rate, stroke volume, contractility, and developed force and pressure during cycles of systole and diastole, which after the exercise overload stimulates compensation and thus adaptation in the recovery and regeneration phase.^12^, ^13^ However, the acute response to the exercise bout, as observed while the exercise is taking place or immediately after, is that the myocardial stress resulting from the increased load leads to transient cardiac fatigue in at least some parameters of systolic and diastolic functions, that is, a temporary cardiac exhaustion,^13^, ^14^, ^15^, ^16^, ^17^, ^18^, ^19^, ^20^, ^21^ and although some studies have indicated this may be more prevalent in the right ventricle (RV),^22^, ^23^, ^24^ others have convincingly shown that this is also a feature of the left ventricle (LV).^13^, ^14^, ^15^, ^16^, ^17^, ^19^, ^20^, ^21^, ^25^ It is thought that this postexercise cardiac depression is caused by reduced cardiomyocyte contractility, SR Ca^2+^ release, and ATP availability,^15^, ^18^, ^26^, ^27^, ^28^, ^29^, ^30^ although mitochondrial ATP generation in some studies has also remained unperturbed following acute exercise.^15^, ^28^, ^31^, ^32^, ^33^ However, these studies face considerable confounding since they apply in vitro techniques to harvest, isolate and prepare tissues or cells *after* completion of acute exercise but *before* they make their measurements. This is because the handling process is lengthy and performed under basal nonbeating conditions that imposes its own stress to the heart due to neurohormonal activation following animal handling and due to the use of transient hypoxia, anesthetics, proteases, collagenases, and Ca^2+^‐free solutions that are necessary for successful isolation and preparation of tissues and cells,^34^, ^35^ all of which together with time itself in non‐exercise conditions may alter the observed outcomes of acute exercise. Similarly, bioengineered muscle preparations have also been utilized, but these derive from and are reminiscent of skeletal and not cardiac muscle preparations^36^ or from cultured human induced pluripotent stem cells that retain cardiac similarities, but ultimately differ from resident cardiomyocytes.^37^

To overcome the difficulties above, we present a different and novel approach. First, an experimental regimen where cardiomyocytes were freshly isolated and prepared before application of acute exercise to the cell itself, was established. A close mimic for acute aerobic endurance exercise was then applied directly to cells‐in‐a‐dish in the form of evoking trains of electrical twitch‐stimulated contractions over prolonged periods of time that replicated acute aerobic endurance exercise sessions. This allowed us to maintain physiologic conditions throughout the experiment, and measure effects *during* and *immediately after* the mimic exercise, whereby the cellular contractile, Ca^2+^, and metabolic responses to acute exercise were investigated.

## 2 MATERIALS AND METHODS

### 2.1 Ethical approval

All experiments were approved by the Institutional Ethics Review Board (University of Glasgow) and carried out in accordance with the UK Animals (Scientific Procedures) Act 1986, which conforms to the Guide for the Care and Use of Laboratory Animals (National Institutes of Health Publication No. 85‐23, revised 1996).

### 2.2 Animals

**Figure 1:**
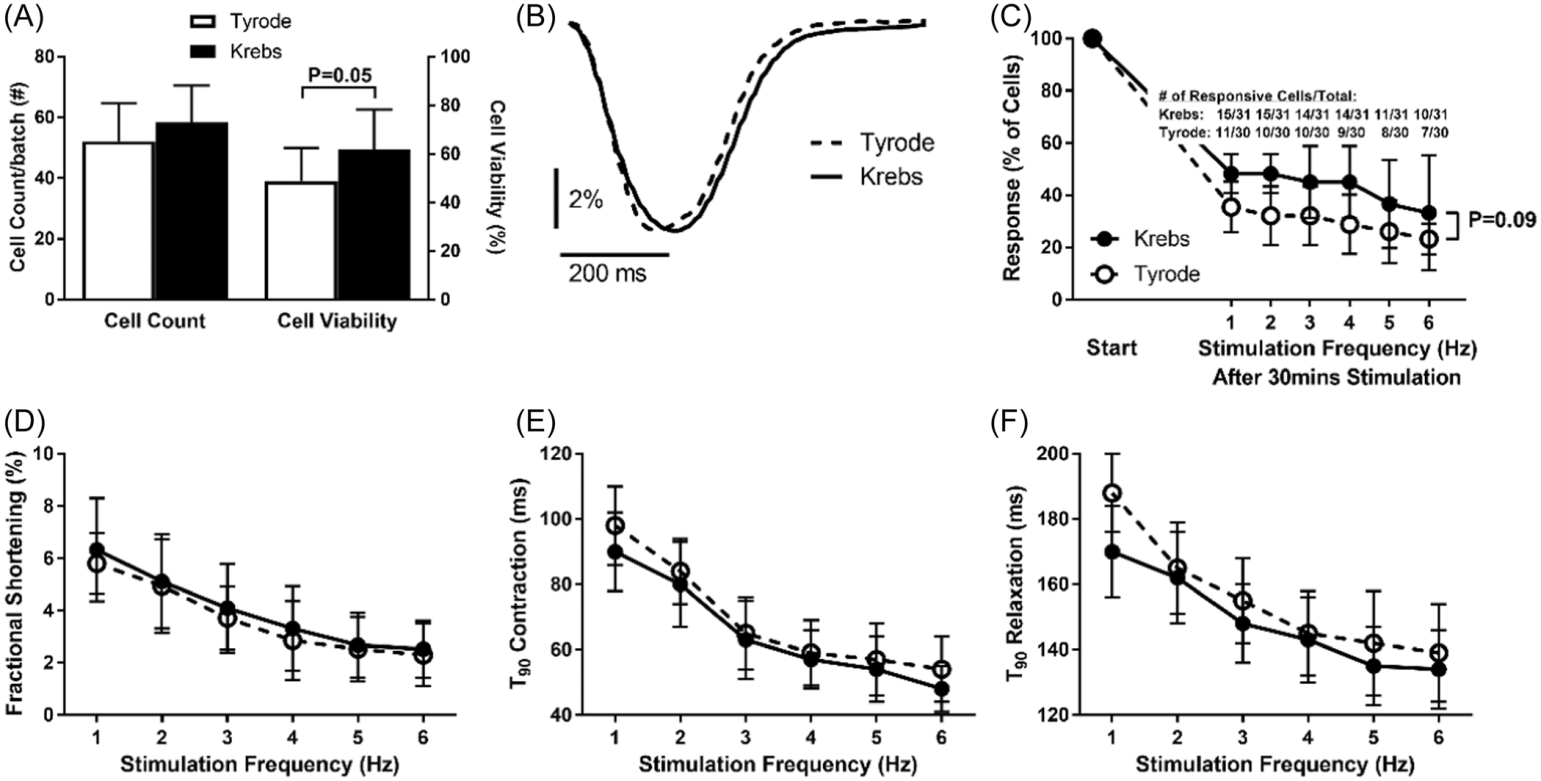
Comparison of cardiomyocyte effects after isolation and incubation in Krebs versus Tyrode solutions (*n* = 6 animals, 4–6 cells/animal for Krebs and Tyrode, respectively, for D–F). (A) Cell count and viability. (B) Example traces of contraction after 1 Hz stimulation for 30 min. (C) Responsive cells after 30 min stimulation (overlay: # of cells responding to the different stimulation frequencies/# of cells that started twitch‐stimulation protocol). (D) Fractional shortening. (E) Time to 90% (*T*_90_) contraction. (F) Time to 90% (*T*_90_) relaxation.

**Figure 2:**
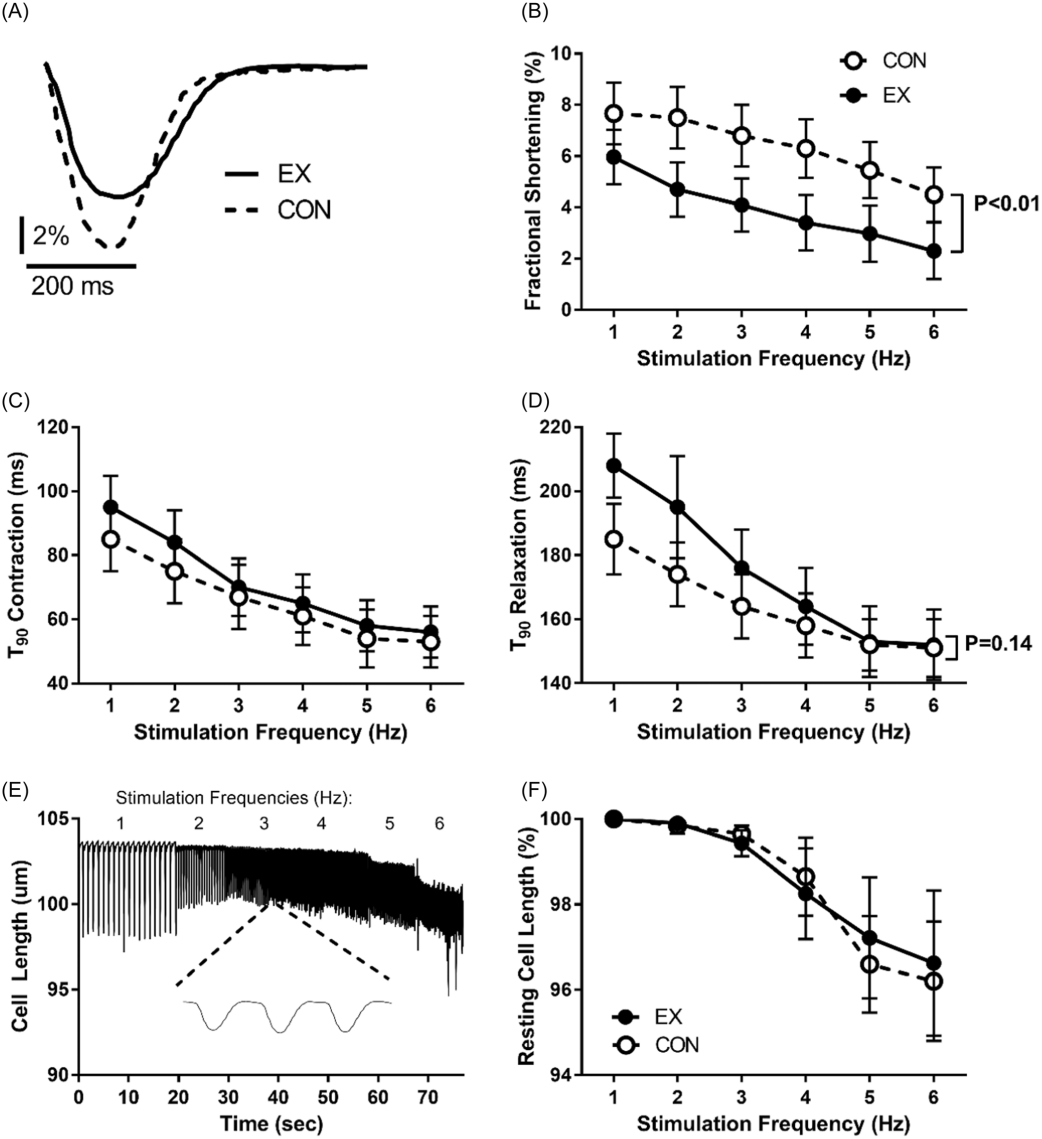
Comparison of contractility after protocols that mimic acute aerobic endurance exercise in perfused cardiomyocytes (Krebs solution, 37°C, pH 7.3, CaCl_2_ 1.8 mM) by applying 30 min trains of 5 ms pulse‐width stimulations at 3 Hz (acute exercise, EX) or control (CON) at 0.3 Hz (*n* = 18 animals, 3–4 cells/animal for EX and CON, respectively). (A) Example traces of contraction–relaxation in acute exercise and control after 30‐min stimulation, measured at 1 Hz stimulation. (B) Fractional shortening. (C) Time to 90% (*T*_90_) contraction. (D) Time to 90% (*T*_90_) relaxation. (E) Example trace of cardiomyocyte length during stimulation 1–6 Hz after acute exercise; insert shows expanded 1 s trace from 3 Hz stimulation. (F) Proportion of resting cell length during postintervention 1–6 Hz stimulation, relative to cell length at 1 Hz stimulation.

**Figure 3:**
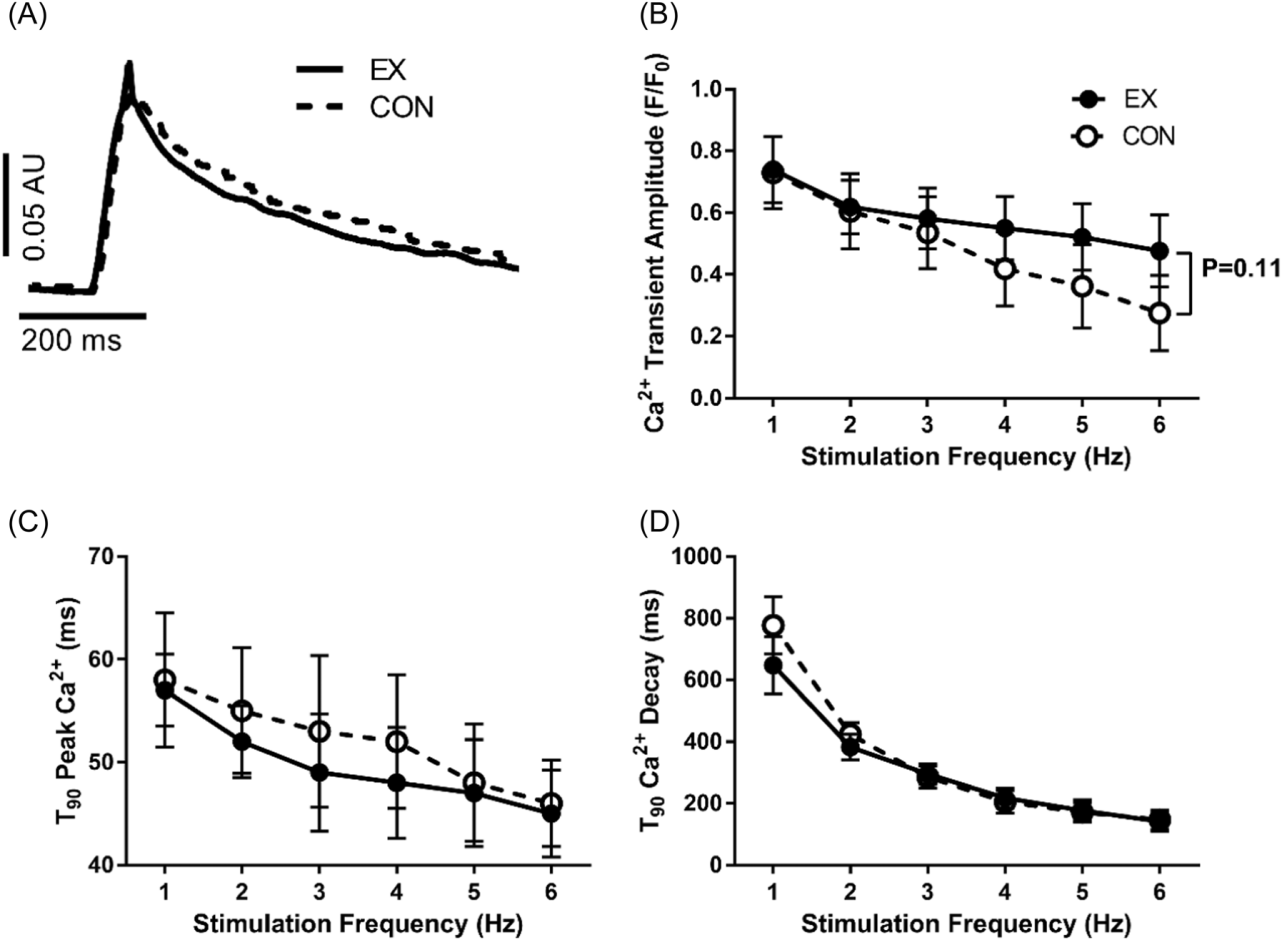
Comparison of intracellular Ca^2+^ handling after protocols that mimic acute aerobic endurance exercise in perfused cardiomyocytes (Krebs solution, 37°C, pH 7.3, CaCl_2_ 1.8 mM) by applying 30 min trains of 5 ms pulse‐width stimulations at 3 Hz (acute exercise, EX) or control (CON) at 0.3 Hz (*n* = 18 animals, 3–4 cells/animal for EX and CON, respectively). (A) Example traces of intracellular Ca^2+^ transients in acute exercise and control after 30‐min stimulation, measured at 1 Hz stimulation. (B) Ca^2+^ transient amplitude. (C) Time to 90% (*T*_90_) peak Ca^2+^. (D) Time to 90% (*T*_90_) Ca^2+^ decay.

**Figure 4:**
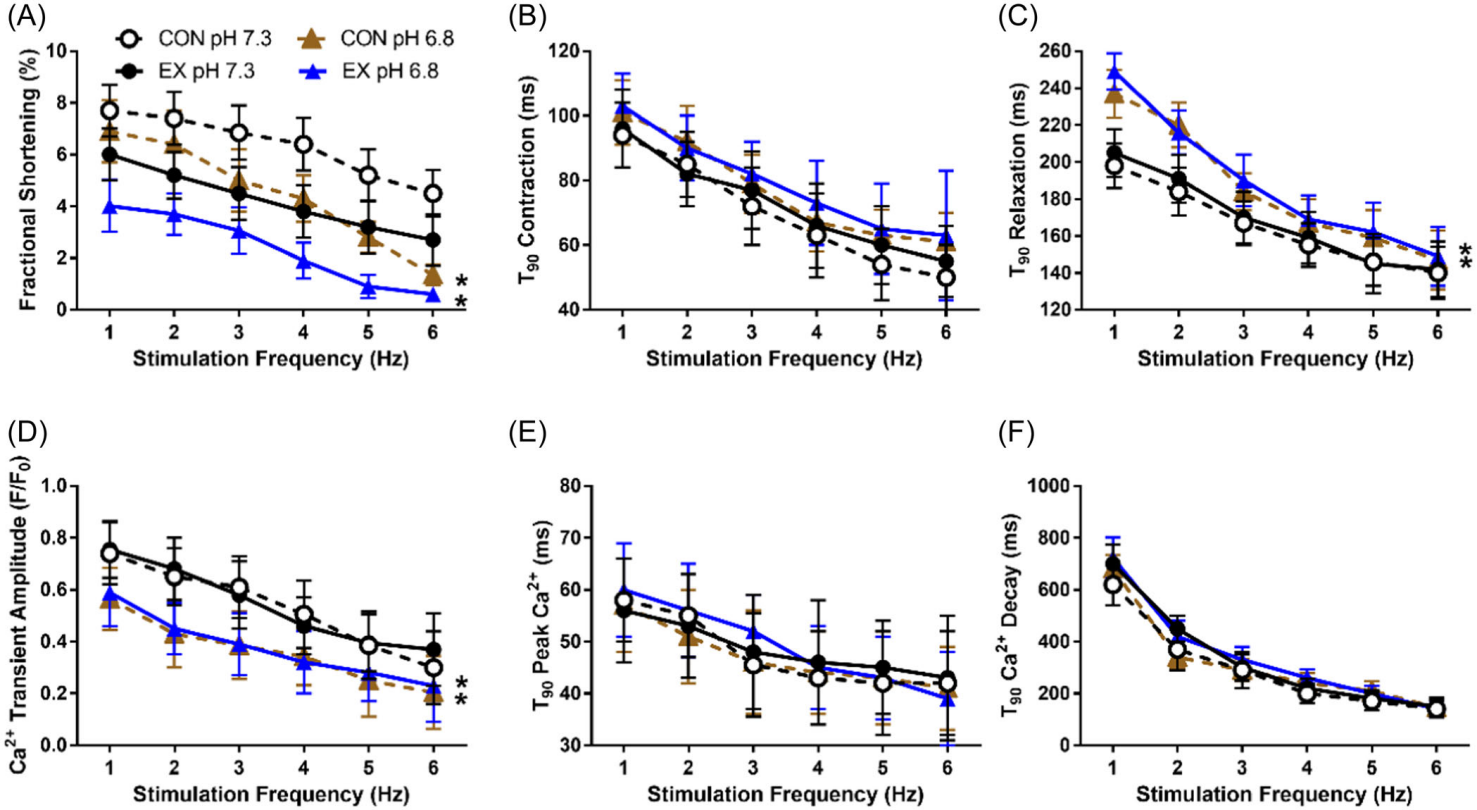
Comparison of contractility and intracellular Ca^2+^ handling after protocols that mimic acute aerobic endurance exercise in perfused cardiomyocytes (Krebs solution, 37°C, pH 7.3, CaCl_2_ 1.8 mM) by applying 30 min trains of 5 ms pulse‐width stimulations at 3 Hz (acute exercise, EX) or control (CON) at 0.3 Hz, with poststimulation measurements recorded at either pH 7.3 or 6.8 (*n* = 6 animals, 3–4 cells/animal for EX and CON at each pH, respectively). (A) Fractional shortening; *p < .05 versus pH 7.3. (B) Time to 90% (*T*_90_) contraction. (C) Time to 90% (*T*_90_) relaxation; *p < .05 versus pH 7.3. (D) Ca^2+^ transient amplitude; *p < .05. (E) Time to 90% (*T*_90_) peak Ca^2+^. (E) Time to 90% (*T*_90_) Ca^2+^ decay.

**Figure 5:**
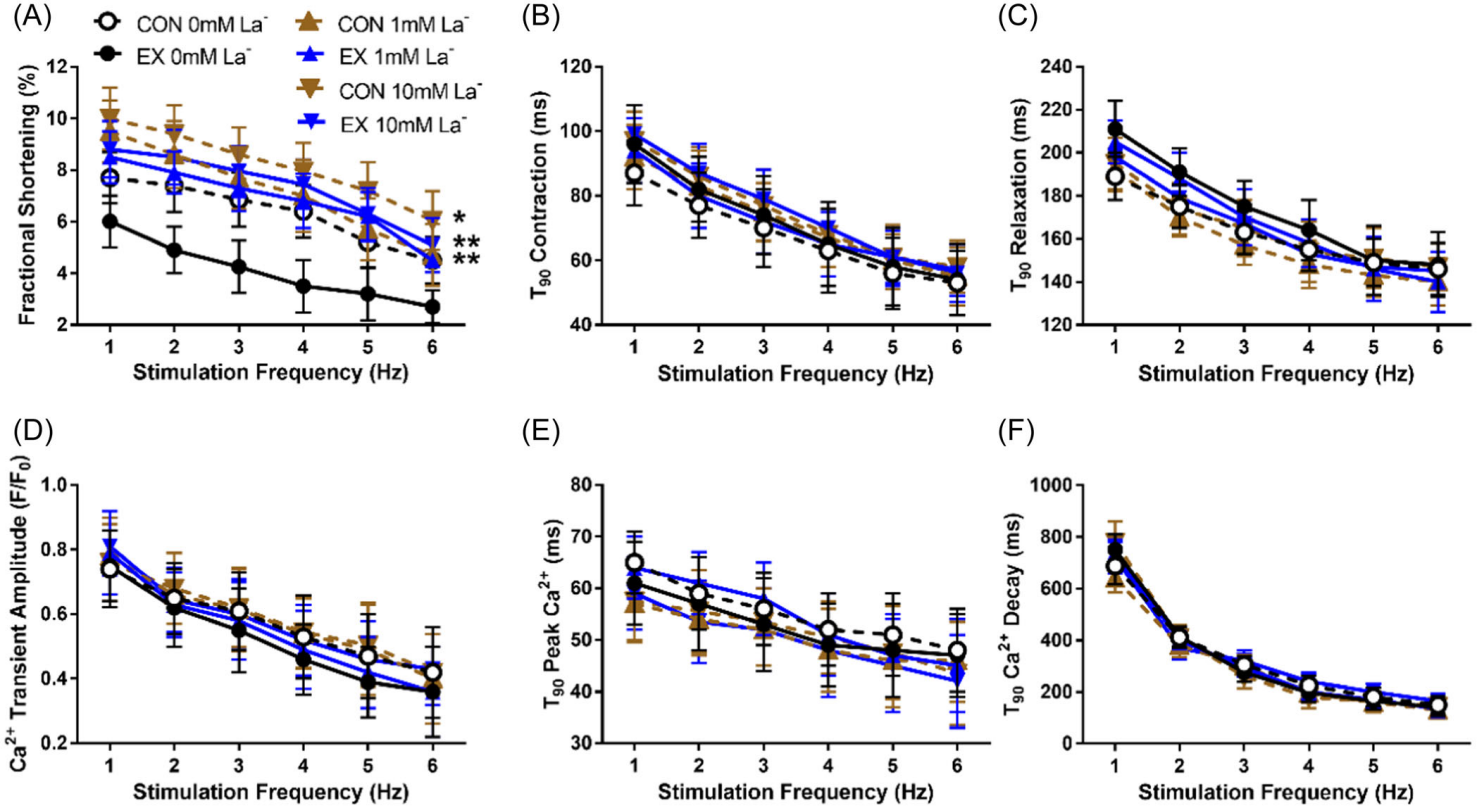
Comparison of contractility and intracellular Ca^2+^ handling after protocols that mimic acute aerobic endurance exercise in perfused cardiomyocytes (Krebs solution, 37°C, pH 7.3, CaCl_2_ 1.8 mM) by applying 30 min trains of 5 ms pulse‐width stimulations at 3 Hz (acute exercise, EX) or control (CON) at 0.3 Hz in the presence of 0 mM, 1 mM, or 10 mM lactate (La^−^, sodium l‐lactate; *n* = 6 animals, 3–4 cells/animal for EX and CON at each La^−^, respectively). (A) Fractional shortening; *p < .05 versus CON 0 mM La^−^, **p < .01 versus EX 0 mM La^−^. (B) Time to 90% (*T*_90_^)^ contraction. (C) Time to 90% (*T*_90_) relaxation. (D) Ca^2+^ transient amplitude. (E) Time to 90% (*T*_90_) peak Ca^2+^. (E) Time to 90% (*T*_90_) Ca^2+^ decay.

**Figure 6:**
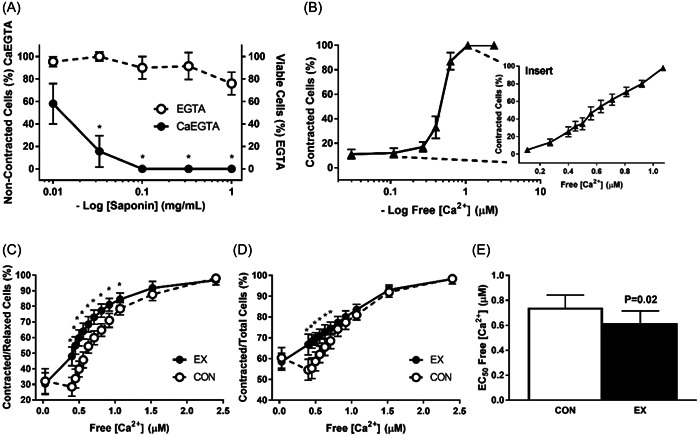
Comparison of intracellular myofilament Ca^2+^ sensitivity after protocols that mimic acute aerobic endurance exercise in cardiomyocytes by applying 30 min trains of 16 ms pulse‐width twitch‐stimulations at 3 Hz (acute exercise, EX) or control (CON) at 0.3 Hz in culture incubators (Krebs solution, 37°C, pH 7.3, CaCl_2_ 1.8 mM). (A) Permeabilization of cardiomyocytes over a range of saponin concentrations ([saponin]) in the presence of free Ca^2+^ (Ca^2+^‐ethylene glycol tetraacetic acid (CaEGTA), whereas absence of free Ca^2+^ (EGTA) indicates nontoxic [saponin]; *p < .05 versus baseline (no saponin) (*n* = 5 animals). (B) Myofilament Ca^2+^ sensitivity over 0.03 µM (10% CaEGTA)–2.40 µM (90% CaEGTA) free Ca^2+^ range to determine [Ca^2+^] that induces contraction (*n* = 5 animals); insert shows more detailed assessment in the range 0.40 µM (60% CaEGTA)–1.07 µM (80% CaEGTA); note *x*‐axis has linear scale (*n* = 5 animals). (C) Index of myofilament Ca^2+^ sensitivity in acute exercise and control after 30 min stimulation, measured by proportion of contracted/relaxed cardiomyocytes; *p < .05 versus CON (*n* = 8 animals). (D) Index of myofilament Ca^2+^ sensitivity in acute exercise and control after 30 min stimulation, measured by proportion of contracted cardiomyocytes/total cells; *p < .05 versus CON (*n* = 8 animals). (E) Free [Ca^2+^] that evoked half‐maximal effect (EC_50_) after acute exercise and control (*n* = 8 animals).

**Figure 7:**
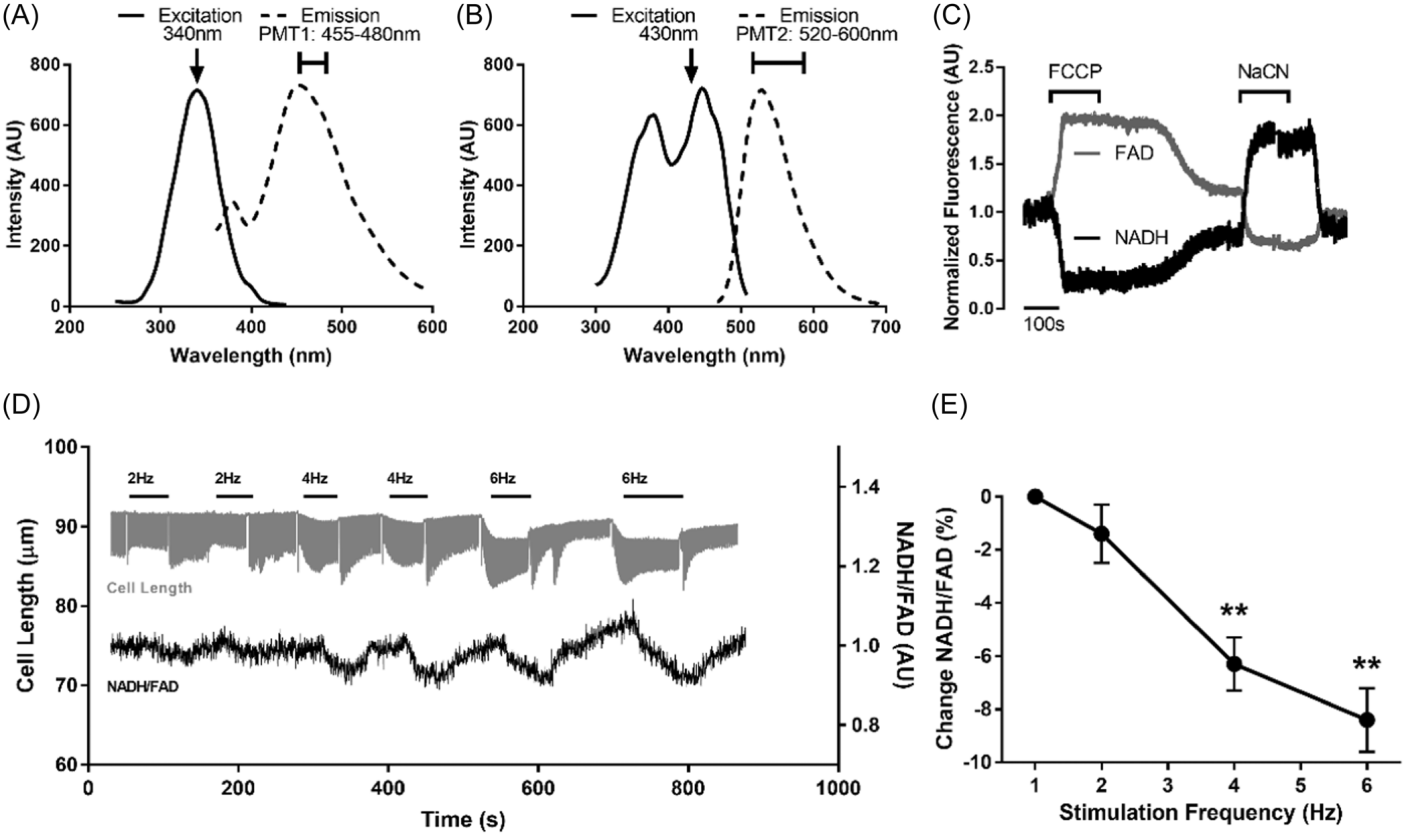
Mitochondrial redox state during protocols that mimic acute aerobic endurance exercise in perfused cardiomyocytes by applying repeated bursts of 5 ms pulse‐width stimulations at progressively increasing frequencies (Krebs solution, 37°C, pH 7.3, CaCl_2_ 1.8 mM), by measuring intrinsic Nicotinamide adenine dinucleotide (reduced, NADH) and Flavin adenine dinucleotide (oxidized, FAD) autofluorescence simultaneously by 2 photomultiplier tubes (PMT). (A) NADH excitation and emission spectra recorded from pure NADH solution as well as intrinsic fluorescence from cardiomyocytes (*n* = 6 animals). (B) FAD excitation and emission spectra recorded from pure FAD solution as well as intrinsic fluorescence from cardiomyocytes (*n* = 6 animals). (C) Example traces of intrinsic NADH and FAD autofluorescence including the effects of 2 µM Carbonyl cyanide *p*‐(trifluoromethoxy)phenylhydrazone (FCCP) and 2 mM Na^+^ cyanide (NaCN) to generate NADH_min_ and FAD_max_, and NADH_max_ and FAD_min_, respectively, with excitation and emission wavelengths as detailed in panels AB for NADH and FAD. (D) Example traces of cardiomyocyte length indicating contraction–relaxation cycles and NADH/FAD ratio during burst electrical field‐stimulation to mimic acute exercise; note progressive decrease to NADH/FAD ratio during increased stimulation frequencies. (E) Changes in autofluorescence NADH/FAD ratio during acute exercise; **p < .01 versus 1 Hz (*n* = 6 animals, 3–4 cells/animal).

We used in total 71 male Wistar rats of 250–300 g (Table 1; Figures 1, 2, 3, 4, 5, 6 and 7C–E) and 6 male New Zealand white rabbits of 3–4 kg (the latter for early‐stage methods development of intrinsic autofluorescence to measure mitochondrial redox state and function for Figure 7A,B); specific *n* numbers for the individual experiments are provided within the Results. Animals were housed with ad libitum water and pellet chow in a 12‐h light‐dark cycle environment. Tissues were shared with other compatible research studies.

### 2.3 Cardiomyocyte isolation and preparation

Chemicals were purchased from Sigma‐Aldrich unless otherwise stated, while fluorescent dyes were purchased from Molecular Probes (Life Technologies).

After cervical dislocation, the heart was excised into ice‐cold Tyrode solution (in mM: 120 NaCl, 20 HEPES, 5.40 KCl, 0.52 NaH_2_PO_4_, 3.50 MgCl_2_, 20 taurine, 10 creatine, 11 glucose [anhydrous]) containing heparin (0.2 mL of 1000 IU/mL) and transferred to retrograde Langendorff perfusion with Tyrode solution containing bovine serum albumin (BSA; 0.1%), type‐2 collagenase (250 IU/mL; Worthington) at 37°C, pH 7.4, for 15 min. The heart was then taken down and LV cardiomyocytes were isolated by dicing, agitating, and filtering in a modified ethylene glycol tetraacetic acid (EGTA)‐containing Krebs solution (in mM: 70 KOH, 40 KCl, 50 l‐glutamic acid, 20 taurine, 20 KH_2_PO_4_, 3 MgCl_2_, 10 glucose [anhydrous], 10 HEPES, 0.5 EGTA) with BSA (1%). After centrifugation at 300 rpm for 2 min and resuspension and incubation in Krebs solution without BSA at 37°C, pH 7.3, for 30 min, cardiomyocytes were stepwise re‐introduced to Ca^2+^ to reach a final suspension of Krebs solution with 1.8 mM CaCl_2_.

### 2.4 Comparison Tyrode and Krebs protocols

The above protocol was arrived at after initial experiments comparing Tyrode and modified EGTA‐containing Krebs solutions for cardiomyocyte isolation and incubation indicated that Krebs better preserved cell viability and contractile function. Specifically, following excision, transfer and retrograde perfusion in Tyrode as described above, we cut the LV in two halves and continued the isolation protocol with each half‐LV in either Tyrode or Krebs solutions as described above, at 37°C, pH 7.3, 30 min, and final CaCl_2_ 1.8 mM, to compare Tyrode versus Krebs solutions for cardiomyocyte isolations, incubations and re‐introductions to Ca^2+^.

First, cell yield and viability was measured by preparing uniform cell suspensions onto a hemocytometer for cell counting on an inverted light microscope (TMS‐F; Nikon) with 10×/0.25 numerical aperture (NA) objective, at room temperature. Next, cardiomyocytes were electrically field twitch‐stimulated at 5ms pulse width, 1 Hz for 30 min (37°C, pH 7.3, CaCl_2_ 1.8 mM), by platinum electrodes connected to a pulse generator and voltage stimulator (Digitimer Train/Delay Generator and Digitimer Constant Voltage Isolated Stimulator, Digitimer Ltd) in a perfusion cell bath mounted on an inverted light microscope (Eclipse‐Ti, Nikon) with 40×/1.3 NA oil‐immersion objective, where responsiveness to stimulation and contractility were recorded via edge‐detection (Myocam‐S, IonOptix) and analyzed (IonWizard 6.1, IonOptix) from 10 stable, consecutive contraction–relaxation cycles after reaching steady‐state at each frequency 1–6 Hz.

### 2.5 Electrical field stimulation as mimic for acute exercise

Previous experience indicates that increasing numbers of cardiomyocytes become non‐contractile when electrical field stimulation train durations and frequencies increase. Therefore, we first quantified this by placing cardiomyocytes in a perfusion cell bath (Krebs solution, 37°C, pH 7.3, CaCl_2_ 1.8 mM) mounted on an inverted light microscope (Eclipse‐Ti, Nikon) with 10×/0.25 NA objective and connected by platinum electrodes to a pulse generator and voltage stimulator (Digitimer Train/Delay Generator and Digitimer Constant Voltage Isolated Stimulator) to induce trains of electrical field 5 ms pulse‐width twitch‐stimulations. From each heart, different batches of cardiomyocytes were stimulated at either of 1, 3, and 6 Hz for 30 min, whereupon we observed numbers of contractile versus noncontractile cardiomyocytes at start and after 10, 20, and 30 min (Table 1), in a design whereby the order of stimulation frequency was balanced to equalize the durations that cells resided in quiescence after isolation but before stimulation. The ×100 magnification allowed us to observe a wide area of the cell bath to increase the number of observations. However, we observed no obvious cell deterioration due to quiescence, but did observe a trend for stimulation time‐dependent decline and a more obvious and significant stimulation frequency‐dependent decline in contracting cells. From these trials, we therefore established the following protocol as a mimic for acute aerobic endurance exercise applied directly to perfused cardiomyocytes in Krebs solution, 37°C, pH 7.3, CaCl_2_ 1.8 mM; applying trains of 5 ms pulse‐width field twitch‐stimulations by platinum electrodes:

− Acute exercise: 3 Hz for 30 min.
− Control: 0.3 Hz for 30 min.

### 2.6 Contractility and intracellular Ca^2+^

Cardiomyocytes were incubated for 12.5 min with 5 µM Fura‐2/Acetoxymethyl ester (AM) in Krebs solution at 37°C, before application of protocols to mimic acute exercise or control in a perfusion cell bath (Krebs solution, 37°C, pH 7.3, CaCl_2_ 1.8 mM) mounted on an inverted light microscope (Eclipse‐Ti, Nikon) with 40×/1.3 NA oil‐immersion objective. Electrical stimulation of 30 min at either 3 or 0.3 Hz of 5 ms pulse‐duration twitch trains were administered by platinum electrodes from a pulse generator and voltage stimulator (Digitimer Train/Delay Generator and Digitimer Constant Voltage Isolated Stimulator). Immediately following the acute exercise mimic or control, stimulation frequency was set to 1 Hz and increased to 6 Hz in 20–30 s steps while simultaneously recording contractile function by edge‐detection (Myocam‐S, IonOptix) and intracellular Ca^2+^ handling by ratiometric epifluorescence excitation at 500 Hz of 340/380 nm light that produced Ca^2+^‐sensitive emission collected at 505–525 nm by a photomultiplier tube (Optoscan, Cairn Research), calibrated for background noise (*F*/*F*_0_). Records of 10 stable, consecutive contraction–relaxation and Ca^2+^‐transient cycles after steady‐state was reached at each stimulation frequency were analyzed for amplitudes and time‐courses (IonWizard 6.1, IonOptix).

### 2.7 Effect of pH and lactate (La^−^)

In different cell batches, we replicated the 30 min acute exercise or control stimulation protocols and subsequent measurements of contractility and intracellular Ca^2+^, first to investigate the effect of acidic conditions by altering pH from normal 7.3 to low pH 6.8 (achieved with HCl) after initial 30 min stimulation at pH 7.3, as pilot experiments indicated that cardiomyocytes did not tolerate 30 min electrical stimulation at pH 6.8. Then, in different cell batches, we investigated the effect of La^−^ (0 mM, 1 mM, or 10 mM sodium l‐lactate) in Krebs solution on contractility and intracellular Ca^2+^.

### 2.8 Myofilament Ca^2+^ sensitivity

First, we assessed tolerable, but effective saponin concentration ([saponin]) for cell permeabilization: We introduced cardiomyocytes to an intracellular solution (in mM: 5 ATP, 10 phosphocreatine [PCr], 100 KCl, 5.50 MgCl_2_, 25 HEPES, 10 EGTA) with a range of [saponin] 0.01–1.00 mg/mL, at room temperature, pH 7.3, for 1 min, followed by centrifugation at 300 rpm for 2 min and resuspension and incubation in intracellular solution without saponin. Cell viability was measured by hemocytometer cell counting (TMS‐F, Nikon with 10×/0.25 NA objective) to identify visibly damaged or dead cells as index of saponin toxicity. This was then repeated, but post‐saponin treatment, intracellular solution contained 10 mM CaEGTA (high Ca^2+^) instead of EGTA, to identify [saponin] that successfully permeabilized cardiomyocytes by counting contracted cardiomyocytes. This indicated 0.10 mg/mL [saponin] was effective and nontoxic for permeabilization and was thus used for subsequent Ca^2+^ sensitivity measurements.

Because this measurement requires bulk quantities of cardiomyocytes, the perfusion cell bath described above was impractical. Acute exercise mimic of 3 Hz or control of 0.3 Hz was therefore applied as trains of 30 V, 16ms pulse‐width field twitch‐stimulations for 30 min in Krebs solution, 37°C, pH 7.3, CaCl_2_ 1.8 mM in culture incubators (C‐Pace, IonOptix), as pilot experiments monitoring the cell chamber (C‐Dish, IonOptix) on an upright stereomicroscope (Nikon SMZ‐2T) with ×60 magnification indicated this protocol to stimulate the largest proportion (70%–80%) of viable cardiomyocytes.

We thus assessed cardiomyocyte myofilament Ca^2+^ sensitivity by measuring contracted:relaxed cell ratios in cardiomyocytes treated with saponin (0.10 mg/mL) via hemocytometry, first in different batches of cells suspended in intracellular solutions of varying EGTA:CaEGTA ratios to ensure different [Ca^2+^], ranging 10% CaEGTA (0.03 µM free Ca^2+^)−90% CaEGTA (2.40 µM free Ca^2+^). This identified the intracellular free [Ca^2+^] that provoked contraction to be in the range 60%–80% CaEGTA (0.40–1.07 µM free Ca^2+^), which was then investigated in more detail by smaller EGTA:CaEGTA ratio steps. Nonlinear curve fitting was used to plot Ca^2+^ sensitivity, from which [Ca^2+^] that evoked half‐maximal contraction effect (EC_50_) was calculated (Origin Pro 9.6, Origin Lab).

### 2.9 Mitochondrial redox state

Reduced NAD (NADH) and oxidized FAD emit intrinsic autofluorescence when excited at ultraviolet (UV) and visible wavelengths, respectively, which permits fluorescence imaging of redox potential and therefore mitochondrial metabolic state.^1^, ^38^, ^39^ When linked with the electrical stimulation protocol, this provides an index of mitochondrial metabolic responses to acute exercise.

Autofluorescence‐based redox potential was measured in perfused cardiomyocytes (Krebs solution, 37°C, pH 7.3, CaCl_2_ 1.8 mM) on an inverted light microscope (Eclipse‐Ti, Nikon) with 40×/1.3 NA oil‐immersion objective by simultaneously exciting NADH and FAD fluorescence at 340 and 430 nm and collecting emission at 455–480 and 520–600 nm bands (Optoscan), respectively. Before this, we verified excitation and emission spectra by widefield epifluorescence spectroscopy (Perkin Elmer LS55, Perkin Elmer) as well as 1‐ and 2‐photon laser scanning microscopy (Zeiss 510 META, Carl Zeiss) in pure NADH and FAD solutions as well as intrinsic fluorescence from cardiomyocytes and applied the mitochondrial inhibitors Carbonyl cyanide *p*‐(trifluoromethoxy)phenylhydrazone (FCCP; 2 µM) to generate NADH_min_ and FAD_max_ and Na^+^ cyanide (NaCN; 2 mM) to generate NADH_max_ and FAD_min_. This allows for calculation of mitochondrial redox state, taken as normalized NAD_state_ (proportion of reduced state NAD: 1.0 = fully reduced, 0.0 = fully oxidized) and FAD_state_ (proportion of oxidized state FAD: 1.0 = fully oxidized, 0.0 = fully reduced), as: (*F*_basal_ − *F*_min_)/(*F*_max_ − *F*_min_) during both resting baseline conditions and acute exercise. However, since prolonged UV radiation creates photodamage that is not sustainable for 30 min,^40^ a modified electrical stimulation protocol to mimic acute exercise was developed. Thus, cardiomyocytes were baseline electrical field‐stimulated at 1 Hz by 5 ms‐pulse‐duration twitch‐stimulation trains (platinum electrodes, Digitimer Train/Delay Generator and Digitimer Constant Voltage Isolated Stimulator), and then we introduced steps of 2 × 50 s bursts of 2, 4, and 6 Hz stimulation, respectively, with recovery at 1 Hz stimulation between steps. At the end of each protocol, NADH and FAD autofluorescence was calibrated as well as confirmed to be of mitochondrial origin by 2 µM FCCP to generate NADH_min_ and FAD_max_ and 2 mM NaCN to generate NADH_max_ and FAD_min_. NADH/FAD ratio was then taken as a mitochondrial oxidation‐sensitive signal, with artefacts and background noise minimized by the ratioing. Cardiomyocyte contractility was also simultaneously recorded by edge‐detection (Myocam‐S).

### 2.10 Statistics

Data are expressed as mean ± SD. Unrelated observations between groups were evaluated by independent samples *t*‐tests and one‐way analysis of variance, or one‐way and two‐way chi‐square (*χ* ^2^) for categorical data, while Repeated measures general linear model evaluated group differences between repeatedly measured variables; Scheffe post hoc tests identified effects where appropriate (SPSS 27, IBM). Significance level was *p* < .05. Cohen's *d* evaluated effect sizes.

## 3 RESULTS

### 3.1 Tyrode versus Krebs

In the first set of experiments, we found that cell isolation and incubation in either of Tyrode or modified EGTA‐containing Krebs solutions resulted in yields of viable cardiomyocytes, but Krebs showed 25% increased cell viability versus Tyrode (Cohen's *d* 0.78 [0.03–1.53]) (Figure 1A). We then subjected cardiomyocytes to 30 min stimulation at 1 Hz, after which stimulation frequency was stepwise ramped up 1–6 Hz, with example traces of contractility at 1 Hz after 30 min stimulation shown (Figure 1B). The number of cardiomyocytes that maintained a contractile response throughout the protocol did not statistically differ between Tyrode and Krebs solutions, but an insignificant trend (*p* = .09) towards increase in Krebs was observed (Figure 1C). In those cardiomyocytes that did preserve a contractile response to the stimulation protocol, all showed reduced magnitudes of fractional shortening (Figure 1D), but increased rates of contractions (Figure 1E) and relaxations (Figure 1F) to increased stimulation frequencies 1–6 Hz; however, no differences were observed between solutions. Because of the trends for increased cell viability and preservation of contractility by Krebs solution, this medium was chosen for subsequent experiments.

### 3.2 Electrical field stimulation as mimic for acute exercise

Next, we measured numbers of cardiomyocytes that were able to respond with intact and faithful contractions during prolonged higher frequency electrical stimulation trains. Increased frequency and train duration of stimulations generally reduced numbers of cardiomyocytes able to contract, but we found that a protocol of 3 Hz stimulation for 30 min was the stimulation protocol with highest frequency and duration that returned an adequately high number of cardiomyocytes fully responding with contractions to the stimulus, whereas 6 Hz was largely intolerable for prolonged durations for the majority of cardiomyocytes and rendered them dysfunctional or inert (Table 1). 3 Hz stimulation for 30 min was therefore used in subsequent experiments as mimic for acute exercise.

**Table 1:** Relative numbers of contracting cardiomyocytes during different protocols.

		Frequency (Hz)		
		1	3	6
Duration (min)	Start	100	100	100
	10	83 ± 6	80 ± 7	32 ± 4**
	20	79 ± 7	74 ± 8	22 ± 7**
	30	61 ± 12	57 ± 10	13 ± 11**

*Note*: Numbers of contracting cardiomyocytes are measured as relative (%) to those that exhibited contractions at start of protocol (*n* = 6 animals). Statistical significance **p < .01 (*χ* ^2^).

### 3.3 Contractility

Based on the above experiments, 30 min of 3 Hz stimulation served as mimic for acute aerobic endurance exercise, whereas control was achieved by 30 min at 0.3 Hz stimulation. This allowed us to study contractile responses to these interventions (examples recordings in Figure 2A). We found that acute exercise decreased fractional shortening throughout the postexercise stimulation frequencies 1–6 Hz 50%–80% (Cohen's *d* 2.41 [1.58–3.34], Figure 2B). In contrast, contraction (Figure 2C) and relaxation (Figure 2D) rates did not change, although a weak insignificant trend towards slowing of relaxation rate was observed after acute exercise.

We also noted that postacute exercise or ‐control, relative resting cell length during 1–6 Hz stimulation decreased (example recording in Figure 2E). This decrease was exaggerated at higher stimulation frequencies (5–6 vs. 1 Hz, *p* < .05 in both acute exercise and control), indicating that at high stimulation‐ and contraction frequencies, the cell does not recover to full relaxation before the subsequent twitch stimulation and signal for contraction arrives and thus is never void of tension during the relaxation phase at high stimulation frequencies; however, no difference between acute exercise and control was observed (Figure 2F).

### 3.4 Intracellular Ca^2+^

A major determinant of cardiomyocyte contractility is intracellular Ca^2+^‐induced Ca^2+^ release. Experimentally this is observed as changes to contractility being preceded by changes to intracellular Ca^2+^. Hence, we assessed cardiomyocyte intracellular Ca^2+^ cycling (example recordings in Figure 3A). In contrast to fractional shortening, release of intracellular Ca^2+^ was not diminished by acute exercise, as no significant change to the amplitude of the Ca^2+^ transient was observed; instead, a weak insignificant trend toward an increase was noted postacute exercise at the higher stimulation frequencies (average effect size Cohen's *d* 0.75 [0.08–1.44], Figure 3B). Rates of Ca^2+^ release (Figure 3C) and Ca^2+^ transient decay (Figure 3D) increased with increasing stimulation frequencies, but did not change with acute exercise.

### 3.5 pH

We repeated acute exercise and control interventions as described above, but this time altering the cell environment from normal pH 7.3 to acidic pH 6.8, after initial 30 min electrical stimulation acute exercise or control at normal pH 7.3. Again, acute exercise depressed fractional shortening (*p* < .01). We further observed that a shift to an acidic cell environment of pH 6.8 by itself depressed fractional shortening, by 1.9 percentage points in both control (Cohen's *d* 1.72 [0.42–3.26]) and acute exercise (Cohen's *d* 1.83 [0.53–3.39]) cardiomyocytes across 1–6 Hz stimulation frequencies; no difference occurred between pH‐induced depressions in control versus acute exercise (*p* > .05), but we noticed a marked accentuation of depression at higher stimulation frequencies, where especially acutely exercised cardiomyocytes displayed very low fractional shortening at 5–6 Hz (Figure 4A). This may have prevented further pH‐dependent reductions at high stimulation frequencies.

Rates of contraction (Figure 4B) did not differ between pH 7.3 and 6.8; however, rates of relaxation were reduced at pH 6.8 versus pH 7.3 by 8%–20% (Cohen's *d* 1.10 [0.01–2.34]) in control cardiomyocytes and by 10%–20% (Cohen's *d* 1.09 [0.09–2.21]) in acutely exercised cardiomyocytes, respectively (Figure 4C).

Similar to above, intracellular Ca^2+^ handling did not differ between control and acute exercise cardiomyocytes (*p* > .05), but the shift to an acidic environment did reduce Ca^2+^ transient amplitude across 1–6 Hz stimulation frequencies, by 20%–30% in both control and acutely exercise (Cohen's *d* 1.28 [0.17–2.55] and 1.25 [0.15–2.50] in control and acute exercise, respectively; Figure 4D). No effects of acidic conditions were observed for rates of rise (Figure 4E) or decay (Figure 4F) of Ca^2+^ transients.

### 3.6 La^−^

We once more repeated the above‐mentioned experiments, but this time in the presence of either no, 1 or 10 mM La^−^. First, these experiments showed that acute exercise decreased fractional shortening across 1–6 Hz stimulation frequencies similar to that reported above (30%–80%, Cohen's *d* 2.12 [1.33–2.91], *p* < .01, Figure 5A). More importantly, when cardiomyocytes were incubated with La^−^, fractional shortening increased in both control and acute exercise; however, this increase was more pronounced after acute exercise versus control: in control cardiomyocytes, no effect was observed in 1 mM La^−^, whereas 10 mM La^−^ increased fractional shortening by 20%–30% (Cohen's *d* 1.62 [0.36–3.09]) across 1–6 Hz stimulation frequencies, whereas after acute exercise, 1 mM La^−^ increased fractional shortening by 40%–90% (Cohen's *d* 2.55 [1.08–4.39]) and 10 mM La^−^ increased fractional shortening by 50%–100% (Cohen's *d* 2.79 [1.26–4.73]) across 1–6 Hz stimulation frequencies and rendered acute exercise not different from control (*p* > .05; Figure 5A). For rates of contraction (Figure 5B) and relaxation (Figure 5C), no effects of La^−^ were observed.

In contrast to the above, no La^−^‐mediated effects were observed for amplitude (Figure 5D) or rates of rise (Figure 5E) or decay (Figure 5F) of Ca^2+^ transients.

### 3.7 Myofilament Ca^2+^ sensitivity

For measuring myofilament Ca^2+^ sensitivity, we first identified the [saponin] that effectively but harmlessly permeabilized cardiomyocytes. In the presence of free Ca^2+^ (CaEGTA), we found that significant, but incomplete permeabilization was achieved at [saponin] 0.03 mg/mL, whereas [saponin] 0.10 mg/mL achieved complete permeabilization, while EGTA experiments indicated that these [saponin] were nontoxic for the permeabilized cardiomyocytes (Figure 6A). Therefore, [saponin] 0.10 mg/mL was used for subsequent cardiomyocyte permeabilization.

The myofilament Ca^2+^ sensitivity assay was initially developed by assessing the range of free [Ca^2+^] from 0.03 µM (10% CaEGTA) to 2.40 µM (90% CaEGTA) in permeabilized cardiomyocytes. This showed that contraction occurred in the free [Ca^2+^] range 0.40 µM (60% CaEGTA) to 1.07 µM (80% CaEGTA; Figure 6B). This range was then studied in more detail with smaller EGTA:CaEGTA steps, which indicated a clear dose–response relationship between intracellular free [Ca^2+^] and myofilament contraction (Figure 6B Insert).

Next, we mimicked acute exercise or control by field‐stimulating cardiomyocytes for 30 min at 3 or 0.3 Hz, respectively (Krebs solution, 37°C, pH 7.3, CaCl_2_ 1.8 mM) in bulk quantities by the use of culture incubators, before permeabilization and introduction to free [Ca^2+^] range 0.03 µM (10% CaEGTA)–2.40 µM (90% CaEGTA), and with detailed scrutiny of the range 0.40 µM (60% CaEGTA)–1.07 µM (80% CaEGTA), as detailed above. This showed that acute exercise increased myofilament Ca^2+^ sensitivity, measured as increased proportion of contracted/relaxed intact cardiomyocytes in acutely exercised cardiomyocytes versus control (Figure 6C), with relative increase of 20%–70% and effect size (Cohen's *d*) ranging 0.84–3.01 across the different free intracellular [Ca^2+^], with the effect especially noticeable at lower intracellular free [Ca^2+^] in the range 0.40–1.07 µM, which is where the majority of the in vivo sarcomeric shortening and cellular contraction occurs.

To eliminate the possibility that decline in viable cardiomyocytes over the measurement period occurred due to spontaneous cell death and thereby introduced confounding, we repeated the measurements, but this time indexing Ca^2+^ sensitivity as proportion of contracted cardiomyocytes/total cells (all cell observations). This confirmed the finding that acute exercise increased myofilament Ca^2+^ sensitivity (Figure 6D). Finally, we calculated free [Ca^2+^] EC_50_, showing that acute exercise decreased EC_50_ 18% versus control (Cohen's *d* 1.09 [0.07–2.21], Figure 6E); that is, half‐maximum effect was reached at lower free [Ca^2+^] after acute exercise, commensurate with increased myofilament Ca^2+^ sensitivity.

### 3.8 Mitochondrial redox state

Finally, cardiomyocyte mitochondrial redox potential as a measure of metabolic state was assessed by intrinsic autofluorescence of reduced state NAD (NADH) and oxidized state FAD. First, we verified NADH and FAD excitation and emission spectra with the use of pure NADH and FAD in separate solutions and then by capturing intrinsic autofluorescence in cells. This indicated that excitation at 340 nm and consequent emission collected at 455–480 generates an NADH‐specific signal, and that excitation at 430 nm and emission collection at 520–600 nm generates an FAD‐specific signal (Figure 7A,B). We then established baseline NAD and FAD states in resting cardiomyocytes from the autofluorescence in the absence and presence of FCCP and NaCN (example recordings in Figure 7C). This yielded baseline resting NAD_state_ of 0.59 ± 0.04 and FAD_state_ of 0.17 ± 0.03, meaning 59% of the NAD pool was in the reduced NADH state (41% oxidized state) and 17% of the FAD pool was in the oxidized state (83% reduced). These results were also confirmed by tunable single‐ and 2‐photon laser scanning microscopy, in which excitation is confined to µm‐scale intracellular loci within the individual cardiomyocyte (data not shown), and altogether confirm that at least the majority of the signal originates from mitochondria.

The above therefore, with the electrical stimulation protocol modified to restrict UV radiation, allowed us to investigate mitochondrial effects of acute exercise as they developed. The contractile response was commensurate with the electrical stimulation protocol and in agreement with our previous observations, whereas NADH autofluorescence decreased, FAD autofluorescence increased, and the resulting NADH/FAD ratio decreased during the progressively increasing stimulation frequency bursts mimicking acute exercise, with returns to baseline in the interim 1 Hz recovery stimulation periods (example recordings in Figure 7D). Thus, we found that NADH/FAD ratio only minimally decreased during 2 Hz stimulation, but decreased to reach values ∼6% and ∼8% below baseline at 4 and 6 Hz stimulation frequencies, respectively (Figure 7E), indicating intracellular oxidation. We also noted that NADH/FAD ratio decreased almost immediately after delivery of high‐frequency stimulation bursts, that is, 50% NADH/FAD autofluorescence change occurred within 10–20 s of increased contractile work.

## 4 DISCUSSION

The heart acutely responds to bouts of endurance exercise training by increasing the rhythm and developed tension of contractions, resulting in increased cardiac output and blood flow. This increase is enabled because cardiomyocytes increase contractile performance.^2^, ^3^, ^41^ However, only elevated cardiac output and blood flow are typically observed, whereas the cellular response is at least partly a supposition, because we have been unable to extract experimental information from the cardiomyocyte during and immediately after bouts of exercise training. The closest we have come has been to exercise a model animal and then subsequently prepare the cell for study, but this adds a lengthy period between the actual exercise and the observed effect and it alters the physiology in question in and of itself by the use of e.g. cardiodepressive anesthetics and solutions,^34^, ^35^ which taken together may mask or disrupt the true cellular responses. Here, we present a different and novel approach that for the first time allows us to study truly acute exercise effects in freshly isolated cardiomyocytes. For this, we established experimental protocols for cells‐in‐a‐dish where step one prepares suitable cardiomyocytes ready for experimental exercise interventions and observations, and step two applies a mimic for acute aerobic endurance exercise directly to either individual or bulk cardiomyocytes. This mimic consists of evoking trains of electrical twitch‐stimulated contractions over prolonged periods of time in a manner that replicates the cardiomyocyte work conditions of acute aerobic endurance exercise sessions, and this thereby allows us to experimentally investigate cellular contractile, Ca^2+^ and metabolic properties *during* and *immediately after* the mimic exercise.

We observed reduced fractional shortening during and immediately after the acute exercise, and with the magnitude of the reduction being accentuated during the highest postexercise stimulation frequencies. We also observed a sustained pattern of incomplete relaxations between contractions. These observations are commensurate with the cardiac fatigue observed in whole hearts during exhaustive exercise^13^, ^14^, ^16^, ^17^, ^19^, ^20^, ^21^; with the observed myocardial stiffness and reduced diastolic function associated with acute exercise,^42^ and with the reduced contractile function observed in cardiomyocytes isolated and prepared after a bout of treadmill running.^15^ Thus, acute exercise substantially compromises cellular contractile ability in the cardiomyocyte, and this effectively explains the previous observations of reduced postexercise contractile functioning and capacity of the heart; that is, cardiac fatigue.

However, the acute exercise‐induced contractile deficit was not caused by changes to intracellular Ca^2+^ handling, which is surprising given that Ca^2+^ handling largely dictates the cardiomyocyte contraction.^1^, ^41^ In fact, we rather observed a weak trend to increased intracellular Ca^2+^ release, especially at higher stimulation frequencies, and moreover, we observed clear myofilament Ca^2+^ sensitization in the intracellular [Ca^2+^] range where the in vivo contraction occurs,^1^ with marked exaggeration in the lowest [Ca^2+^] where early‐phase contraction occurs, following acute exercise. Thus, acute changes to intracellular availability and myofilament sensitivity to Ca^2+^ appears to mitigate and compensate for the contractile deficit, especially where it matters for contraction. It seems reasonable to suppose that without the upregulated Ca^2+^ effects, contractile function may have been even further reduced, and that this mitigation and compensation most likely contributes to preserve whole‐heart function during exercise in a manner that permits for continued exercise at a higher effort level. This is in contrast to previous reports,^15^, ^18^, ^43^ but those studies used a protocol where they observed the exercise effect long after the exercise was carried out.

So what does limit cardiomyocyte fractional shortening during acute exercise, if not Ca^2+^? Intracellular pH and acute exercise‐associated acidification of the intracellular environment may be one cause, as previous studies have shown that intracellular pH falls ∼0.5 pH units (from 7.3 to 6.8) during intense electrical stimulation bouts comparable to those in our study and that this associates with reduced contractions.^8^ We did not measure intracellular pH per se, but instead, we evoked this acidification and observed substantial reductions in cardiomyocyte intracellular Ca^2+^ release, contractions and relaxations, which were accentuated by high‐frequency stimulations. Hence, if acute exercise reduces intracellular pH in the cardiomyocyte, as observed elsewhere,^8^ reduced intracellular Ca^2+^ release and cellular contraction will follow. This may be explained by competitive H^+^‐binding to troponin that consequently decreases Ca^2+^‐binding and therefore disrupts Ca^2+^‐induced activation of contraction and by disruption of Ca^2+^‐independent myofilament mechanical properties,^44^ which our results support by the observation that intracellular acidification had a greater suppressive effect on contractile function than Ca^2+^ cycling.

However, the bigger impact may be caused by a metabolic mismatch between ATP demand and supply. Acute exercise is energy‐demanding, and our next experiments in the presence of La^−^ indicated that increased energy supply mitigated the contractile deficiency induced by acute exercise; in fact, we observed that contractile performance increased in both acutely exercised and control cardiomyocytes in a La^−^ concentration‐dependent manner. This was more pronounced after acute exercise, to the degree that the previously observed difference between acute exercise and control disappeared after application of La^−^, and moreover, we observed that contractile performance increased more with 10 mM La^−^ than 1 mM. This is not surprising since La^−^ facilitates mitochondrial respiration,^45^ and it indicates that the acute exercise‐induced contractile reduction may be caused by inadequate mitochondrial ATP generation.

To investigate this possibility in more depth, we established protocols for measuring intrinsic NADH/FAD autofluorescence and isolated the signal to the mitochondria by the use of FCCP and NaCN, as this provides an index of redox potential,^38^, ^39^ and which together with simultaneous measures of contractile performance links metabolism to workload; all during incrementally and progressively increasing bursts of electrical stimulation mimics of acute exercise. These experiments showed that acute exercise induced an immediate decrease in NADH/FAD ratio, which indicates oxidation of the cardiomyocyte intracellular environment.^38^, ^39^ This suggests that energy supply is not matching demand upon transitioning from rest to exercise, especially during increased contractile workloads, and once workload eased, the mismatch reversed. Thus, the cardiomyocyte is metabolically challenged with compromised mitochondria in which the production of reducing equivalents by the tricarboxylic acid cycle is not well matched to the increased rate of consumption, and therefore the mitochondria are unable to generate adequate ATP to appropriately fuel the acute exercise, at least under the present experimental conditions. This effectively limits cardiomyocyte contractile capacity and performance during acute exercise, and it therefore also remains possible that if the cardiomyocyte was offered adequate mitochondrial substrate, fatigue might have been delayed and contractile function preserved. Nonetheless, our results of redox state at rest, corresponding to State 3 respiration in isolated mitochondria and characterized by aerobic conditions with adequate substrate,^46^ and although the FAD pool was in a more reduced state than NAD, this suggests that the cardiomyocyte in baseline resting conditions has a high reserve capacity for aerobic respiration, which may be taxed during stress situations such as acute exercise. Both the redox potential at rest^47^ and changes occurring during acute exercise including oxidation of the intracellular environment have also been previously observed, in response to other metabolic challenges^38^, ^39^ or in response to increased stimulation frequencies in cells^48^ or isolated trabeculae.^49^ One interpretation of this may be increased oxidative stress,^49^ but it is also possible that this effect constitutes part of the physiologic response to acute exercise in a manner that also includes activation of antioxidant defense as protection against oxidative stress and that thereby facilitates for the increased cardiac performance during acute exercise.^50^

### 4.1 Strengths and limitations

The present cell culture mimic of acute exercise studies the intrinsic response of the isolated cardiomyocyte to that mimic. However, the current protocols exclude extracellular regulation and influence of mechanical loading from the normal in vivo environment in the heart,^51^, ^52^ as well as contributions of other cell types,^53^ all of which also contribute to in vivo exercise responses. In practice, this leads to profound perturbation to electrical and mechanical stability, which means that electrical stimulation frequencies differ from physiologic heart rates (in vitro 3 Hz‐180 beats/min vs. in vivo >5 Hz‐300beats/min) and contractions are unloaded with no external mechanical stress. Thus, not all aspects of cellular contractions are recapitulated in our model; however, contraction–relaxation cycling is generated, modulated, and fueled by intracellular contractile, Ca^2+^, and metabolic processes similar to in vivo conditions.^1^, ^41^ Moreover, this study investigates cardiomyocytes originating from the rat left ventricle. In human hearts, the possibility has been raised that acute exercise may exert even greater effects to the RV.^22^, ^23^, ^24^ Thus, the current approach provides insight into the acute intracellular process of the cardiomyocyte during and immediately after exercise, which other experimental techniques have difficulty with.

The current stimulation protocol mimics aerobic endurance exercise, and the observed metabolic alterations link well with those expected following aerobic endurance exercise.^32^, ^50^ Although anaerobic energy processes may contribute in this scenario, they would not be challenged. To replicate anaerobic exercise, a mimic would likely require higher stimulation frequencies over shorter durations and possibly mechanical loading of the cell.

Moreover, each cell that has been quantified has also observably responded appropriately to the electrical stimulation, rendering a high confidence in the cellular stimulus‐response relationship. We initially piloted experiments with electrical stimulation of bulk quantities of cardiomyocytes in culture incubators to increase the cell numbers, but this was discarded because a small fraction of cells would not respond to electrical stimulation; however, for the Ca^2+^ sensitivity assay, bulk quantities were required.

## 5 CONCLUSIONS

We developed a novel experimental in vitro approach to study the acute cellular and intracellular responses to exercise in the heart, whereby cardiomyocytes were freshly isolated and prepared before subjecting them to a mimic for exercise stress, delivered by exercise session‐length trains of electrical twitch‐stimulations that thence replicated an aerobic endurance exercise session. This showed that *during* and *immediately after* the acute exercise: (1) cellular contraction is reduced, (2) which was not caused by intracellular Ca^2+^ handling, (3) rather, intracellular Ca^2+^ handling compensated for contractile decline by upholding Ca^2+^ release and cycling and by myofilament Ca^2+^ sensitization, (4) if intracellular pH is not maintained, this contributes to contractile decline, and (5) lack of energy substrate and mitochondrial inability to match ATP generation to demand strongly contributes to contractile decline. Hence, we identify cell physiologic factors that during acute exercise fail to maintain a normal functioning, and other factors that mitigate for this dysfunction.

## CONFLICT OF INTEREST STATEMENT

The authors declare no conflict of interest.

## Data Availability

The data that support the findings of this study are available in the article and from the corresponding author upon reasonable request.
